# Comparison of Growth of *Borrelia afzelii*, *Borrelia garinii*, and *Borrelia burgdorferi* Sensu Stricto at Five Different Temperatures

**DOI:** 10.1371/journal.pone.0157706

**Published:** 2016-06-16

**Authors:** Gorana Veinović, Eva Ružić-Sabljić, Franc Strle, Tjaša Cerar

**Affiliations:** 1 Department of Microbiology and Immunology, Faculty of Pharmacy, University of Belgrade, Belgrade, Serbia; 2 Institute of Microbiology and Immunology, Faculty of Medicine, University of Ljubljana, Ljubljana, Slovenia; 3 Department of Infectious Diseases, University Medical Centre Ljubljana, Ljubljana, Slovenia; University of Kentucky College of Medicine, UNITED STATES

## Abstract

Lyme borreliosis is caused by the spirochete *Borrelia burgdorferi* sensu lato, a fastidious bacterium that replicates slowly and requires special conditions to grow in the laboratory. Borrelia isolation from clinical material is a golden standard for microbiological diagnosis of borrelial infection. Important factors that affect *in vitro* borrelia growth are temperature of incubation and number of borrelia cells in the sample. The **aim** of the study was to assess the influence of temperature on borrelia growth and survival by evaluation and comparison of growth of 31 different borrelia strains at five different temperatures and to determine the influence of different inoculums on borrelia growth at different temperatures. Borreliae were cultured in the MKP medium; the initial and final number of spirochetes was determined by dark field microscopy using Neubauer counting chamber. The growth of borrelia was defined as final number of cells/mL after three days of incubation. For all three *Borrelia* species, the best growth was found at 33°C, followed by 37, 28, and 23°C, while no growth was detected at 4°C (*P<*0.05). The growth of *B*. *afzelii* species was weaker in comparison to the other two species at 23, 28, 33 and 37°C (*P*<0.05), respectively. There was no statistically significant difference between the growth of *B*. *garinii* and *B*. *burgdorferi* sensu stricto at 28, 33, and 37°C (*P*>0.05), respectively. Inoculum had statistically significant influence on growth of all three *Borrelia* species at all tested temperatures except at 4°C.

## Introduction

Lyme borreliosis is a multisystem disease caused by the spirochetes of the *Borrelia burgdorferi* sensu lato complex that are transmitted by the hard ticks of the *Ixodes* species complex [1, 2].

In Europe, at least four *Borrelia* species (*B*. *afzelii*, *B*. *garinii*, *B*. *burgdorferi* sensu stricto, *B*. *spielmanii*) can cause disease in humans, but some other species have also been reported to be a rare (*B*. *bissettii* and *B*. *lusitaniae*) or potential (*B*. *valaisiana*) causes of human disease. In contrast, *B*. *burgdorferi* sensu stricto is the only known species that cause human disease in North America [1, 3, 4].

Spirochetes can be isolated from skin, blood, cerebrospinal fluid (CSF), and other clinical materials during early as well as late Lyme borreliosis [1, 5, 6]. The clinical material for isolation should be transported to the laboratory as soon as possible; if feasible, specimens such as skin and CSF, must be inoculated immediately into the culture medium. Isolation, as well as cultivation are demanding procedures that are performed in a limited number of laboratories [6, 7].

Borreliae are fastidious, slow-growing, and biochemically inactive bacteria that need special care and optimal conditions for growth such as anaerobic environment and temperature between 30 and 34°C [8, 9]. Some borrelia strains grow well also at higher temperatures (35–39°C), but temperature ≥ 40°C substantially reduce or prevent their growth [10–13]. Generally, low temperatures (room or lower) are better tolerated than high temperatures (37–42°C) [9].

The *in vitro* generation time of borrelia ranges from 7 to 20 hours; it is influenced by available nutrients, conditions of cultivation and adaptation of borrelia to the artificial medium [9] Cultivation from clinical material may last up to 12 weeks, which is much longer than for the majority of other human bacterial pathogens [5, 7, 14]. Borrelia requires complex liquid media for *in vitro* cultivation, due to inability to synthesize any amino acids, nucleosides, nucleotides, fatty acids, and severalother cellular building blocks [15]. For a routine laboratory work, modified Kelly-Pettenkofer (MKP), Barbour-Stoenner-Kelly II (BSK-II) and commercially available BSK-H (Sigma, USA) are the most commonly used media [10, 16, 17].

In addition, temperature during clinical material transportation from patient to the laboratory is important for borrelia survival. Room temperature was reported as suitable for transport of samples infected with borrelia during the period from one to 11 days, while refrigerator temperature (5°C) was described as inadequate [18–20].

The **aim** of the study was to assess and compare the growth of *B*. *afzelii*, *B*. *garinii*, and *B*. *burgdorferi* sensu stricto strains at five different temperatures (4, 23, 28, 33, and 37°C) and to examine the influence of different inoculum on the growth at different temperatures.

## Materials and Methods

### Borrelia strains

Thirty-one strains, 10 *B*. *afzelii*, 10 *B*. *garinii*, and 11 *B*. *burgdorferi* sensu stricto were randomly selected from the collection of strains of the Institute of Microbiology and Immunology, Faculty of Medicine, University of Ljubljana, Slovenia. Isolates were obtained from 31 patients diagnosed with different clinical manifestations of Lyme borreliosis at the Department of Infection Diseases of the University Medical Center Ljubljana, Slovenia. *Borrelia* species of the isolated strains was determined by MluI-restriction fragment length polymorphism (Mlu-RFLP) as described previously [5, 21, 22]. Data regarding the origin of strains are listed in Table 1. Stock cultures of these low passage isolates had been stored at –80°C; for the study, we inoculated and cultured them in the MKP medium [5, 23].

**Table 1:** Origin of *Borrelia afzelii*, *Borrelia garinii*, and *Borrelia burgdorferi* sensu stricto strains analyzed in the study.

*Borrelia afzelii*		*Borrelia garinii*		*Borrelia burgdorferi* sensu sticto	
Strain number	Source	Strain number	Source	Strain number	Source
941/08	Skin	468/08	Skin	2249/07	Skin
819/08	Skin	1881/08	Skin	851/97	Skin
1131/08	Skin	1291/06	Skin	2093/06	Skin
1539/08	Skin	2507/06	Skin	1506/08	Skin
2357/08	Skin	531/08	CSF	2130/06	Skin
999/09	Skin	553/08	CSF	2213/06	Skin
884/09	CSF	892/08	CSF	1963/06	Skin
2498/08	CSF	932/09	CSF	2157/06	Skin
2588/03	Blood	13745/05	CSF	1442/99	CSF
1170/09	Blood	1459/09	CSF	953/03	Blood
				2092/06	Blood

CSF = cerebrospinal fluid.

### Cultivation and numbering of spirochetes

The study was carried out in aseptic conditions provided by laminar flow box in order to reduce the risk of contamination. An aliquot of frozen spirochetes was thawed at room temperature, transferred into starting tubes with 7 mL of MKP medium and incubated at 33°C. Borrelia strains were allowed to multiply to cell density of about 10^6^/mL (4-day-old cultures, with a cell motility >95%) that was determined by dark filed microscopy using Neubauer counting chamber.

The study was conducted using starting tubes with borrelia cells/mL in the range: 1x10^5^-2.5x10^7^ for *B*. *afzelii*, 3x10^5^-5x10^7^ for *B*.*garinii*, and 8.5x10^5^-4.8x10^7^ for *B*. *burgdorferi* sensu stricto strains. Within the range of individual species different concentrations of borrelia cells/mL were distinguished and further processed; generally, these concentrations were reported as low and high. Experiment with individual borrelia strain at particular temperature was repeated at least 10 times with low and high concentration, respectively.

After establishing the exact number of cells/mL in starting tubes, we inoculated aliquots of 50 μL into five new tubes with 7 mL of fresh MKP medium and determined the initial number of cells/mL in inoculated tubes. The tubes were incubated at five different temperatures: 4, 23, 28, 33 and 37°C. After 3 days of incubation we checked the number of spirochetes (final number). The initial and the final number of spirochetes were determined by dark filed microscopy using Neubauer counting chamber. Growth was defined as an increase in the number of cells/mL comparing the initial number and the number of cells/mL after three days of incubation (final number).

### Statistical analysis

All statistical tests were performed using SigmaPlot 11.0 (Systat. Software Inc., Richmond, CA, USA). All graphics were created using MS Excel 2007.

Correlation between the initial and final number of borrelia cells/mL at particular temperature was assessed using Spearman’s rho correlation coefficient. A linear regression analysis was applied to evaluate the influence of initial number of cells/mL on the growth of borrelia.

Kruskal-Wallis and Mann-Whitney tests were used to compare growth of *Borrelia* species at five and two different temperatures, respectively. Values were expressed as median, 25th percentile (P25) and 75th percentile (P75).

*P* values less than 0.05 were considered statistically significant to all statistical analyses.

### Ethics Statement

The study was approved by the National Medical Ethics Committee of the Republic of Slovenia (No: 35p/10/12).

## Results

Results of the present study are based on the growth analysis of 31 different borrelia strains at five different temperatures.

### Comparison of the influence of the initial number (inoculum) on Borrelia growth

Using Spearman’s rho correlation coefficient we found statistically significant positive correlation between the initial and final number for all three *Borrelia* species cultivated at 23, 28, 33 and 37°C, respectively, but not for the strains cultivated at 4°C (Table 2).

**Table 2:** Relationship between the initial and final number of borrelia cells/mL (*Borrelia afzelii*, *Borrelia garinii*, and *Borrelia burgdorferi* sensu stricto) assessed by Spearman’s rho correlation coefficient.

**Temperature**	Initial/final number* **of *B*. *afzelii* strains/mL (rho)**	***P* value**
**4°C**	0.105	**0.291**
**23°C**	0.514	< 0.001
**28°C**	0.381	< 0.001
**33°C**	0.551	< 0.001
**37°C**	0.110	0.019
**Temperature**	Initial/final number* **of *B*. *garinii* strains/mL (rho)**	***P* value**
**4°C**	0.154	**0.127**
**23°C**	0.329	0.001
**28°C**	0.294	0.003
**33°C**	0.454	< 0.001
**37°C**	0.269	0.007
**Temperature**	Initial/final number* **of *B*. *burgdorfer i*sensu stricto/mL (rho)**	***P* value**
**4°C**	0.052	**0.585**
**23°C**	0.434	< 0.001
**28°C**	0.446	< 0.001
**33°C**	0.460	< 0.001
**37°C**	0.330	< 0.001

*Growth was defined as an increase of the number of cells comparing the initial and the final number of cells/mL after three days of incubation at individual temperature (4, 23, 28, 33, and 37°C) presenting as Spearman’s rho correlation coefficient.

Results of linear regression showed that the initial number of cells/mL had statistically significant influence on the growth of all three *Borrelia* species at 23, 28, 33 and 37°C, but not at 4°C. With the initial number of *B*. *afzelii* cells/mL 0.8% [adjusted (R^2^ 0.008)], 23% (R^2^ 0.230), 14.8% (R^2^ 0.148), 34.5% (R^2^ 0.345), and 12% (R^2^ 0.12) of growth at 4, 23, 28, 33, and 37, respectively, can be explained. The corresponding findings for *B*. *garinii* were 0.2% [adjusted (R^2^ 0.002)], 9.7% (R^2^ 0.097), 6.8% (R^2^ 0.068), 27.4% (R^2^ 0.274), and 7.8% (R^2^ 0.078), respectively; and for *B*. *burgdorferi* sensu stricto 0.4% [adjusted (R^2^ 0.004)], 12.7% (R^2^ 0.127), 15% (R^2^ 0.150), 10.7% (R^2^ 0.107), and 3% (R^2^ 0.03), respectively.

Moreover, positive β coefficient indicated a statistically positive relationship between the initial and final number of cells/mL for all three *Borrelia* species growing at 23, 28, 33 and 37°C, but not at 4°C. Results are presented in Table 3.

**Table 3:** Influence of the initial number of cells/mL (inoculum) on growth of *Borrelia afzelii*, *Borrelia garinii*, and *Borrelia burgdorferi* sensu stricto strains at five different temperatures (4, 23, 28, 33, and 37°C).

Species	Dependent variable* Growth** at	F-value	*P*-value	AdjustedR^2^	Standardized Coefficient (β)	*P*-value
*B*. *afzelii*	4°C	0.16	0.685	0.008	0.040	0.685
	23°C	31.15	<0.001	0.230	0.487	<0.001
	28°C	18.48	<0.001	0.148	0.395	<0.001
	33°C	50.51	<0.001	0.345	0.593	<0.001
	37°C	12.45	0.012	0.12	0.289	0.012
*B*. *garinii*	4°C	1.165	0.281	0.002	0.109	0.281
	23°C	11.64	0.001	0.097	0.326	0.001
	28°C	8.34	0.004	0.068	0.278	0.004
	33°C	35.76	<0.001	0.274	0.531	<0.001
	37°C	9.27	0.003	0.078	0.295	0.003
*B*.*burgdorferi* sensu stricto	4°C	0.53	0.464	0.004	0.070	0.464
	23°C	17.02	< 0.001	0.127	0.368	< 0.001
	28°C	20.46	< 0.001	0.150	0.398	< 0.001
	33°C	13.74	< 0.001	0.107	0.340	< 0.001
	37°C	4.36	0.039	0.03	0.198	0.039

*Dependent variable is the final number of borrelia cells/mL. Independent variable is the initial number of borrelia cells/mL at particular temperature.

**Growth was defined as an increase of the number of cells comparing the initial and final number of cells/mL after three days of incubation at five different temperatures (4, 23, 28, 33, and 37°C)

### Growth of strains belonging to individual *Borrelia* species at different temperatures

**Fig 1:**
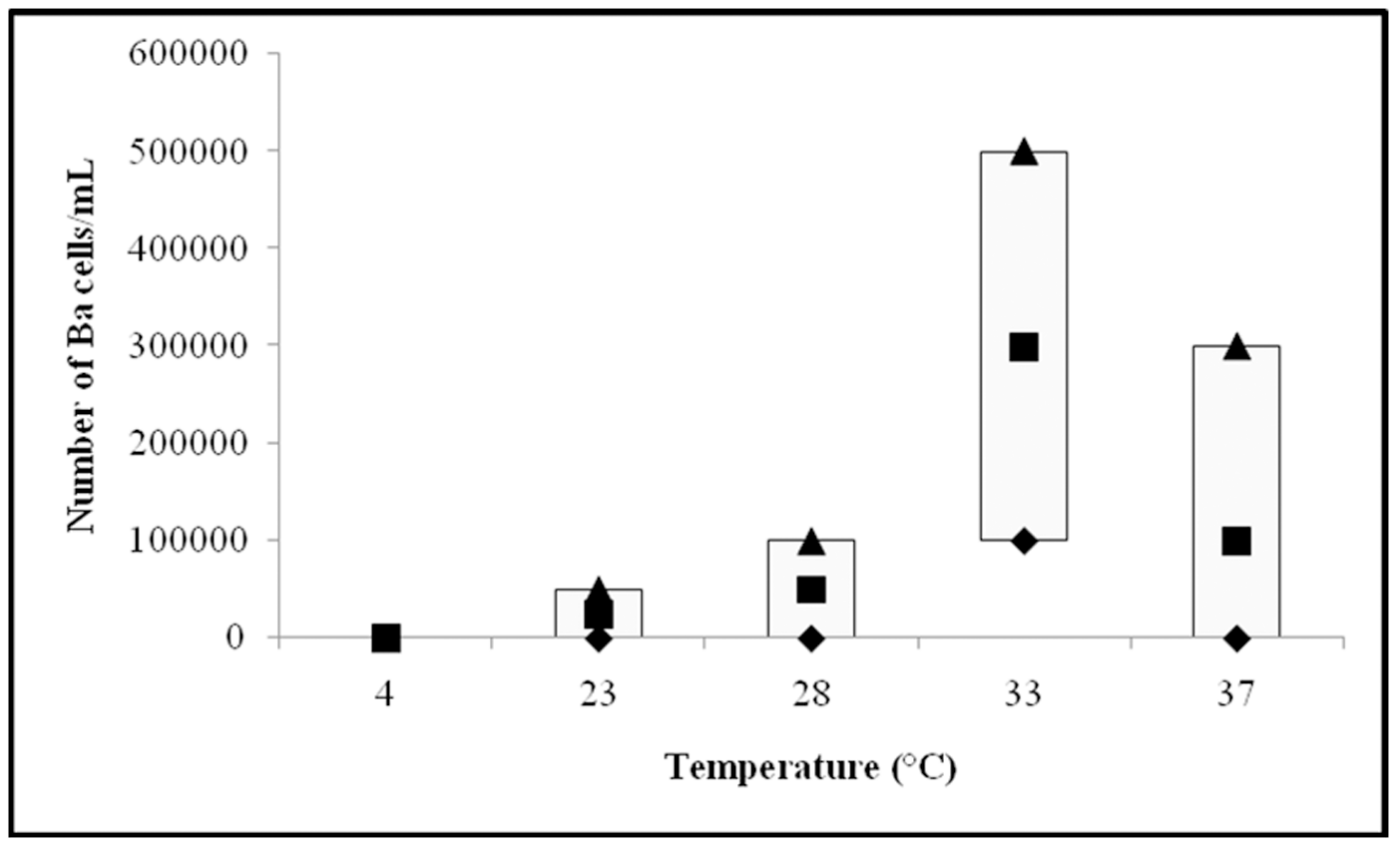
Growth of *Borrelia afzelii* (Ba) strains at 4, 23, 28, 33, and 37°C. Median (■), P25 (♦) and P75 (▲) are expressed as number of cells/mL.

**Fig 2:**
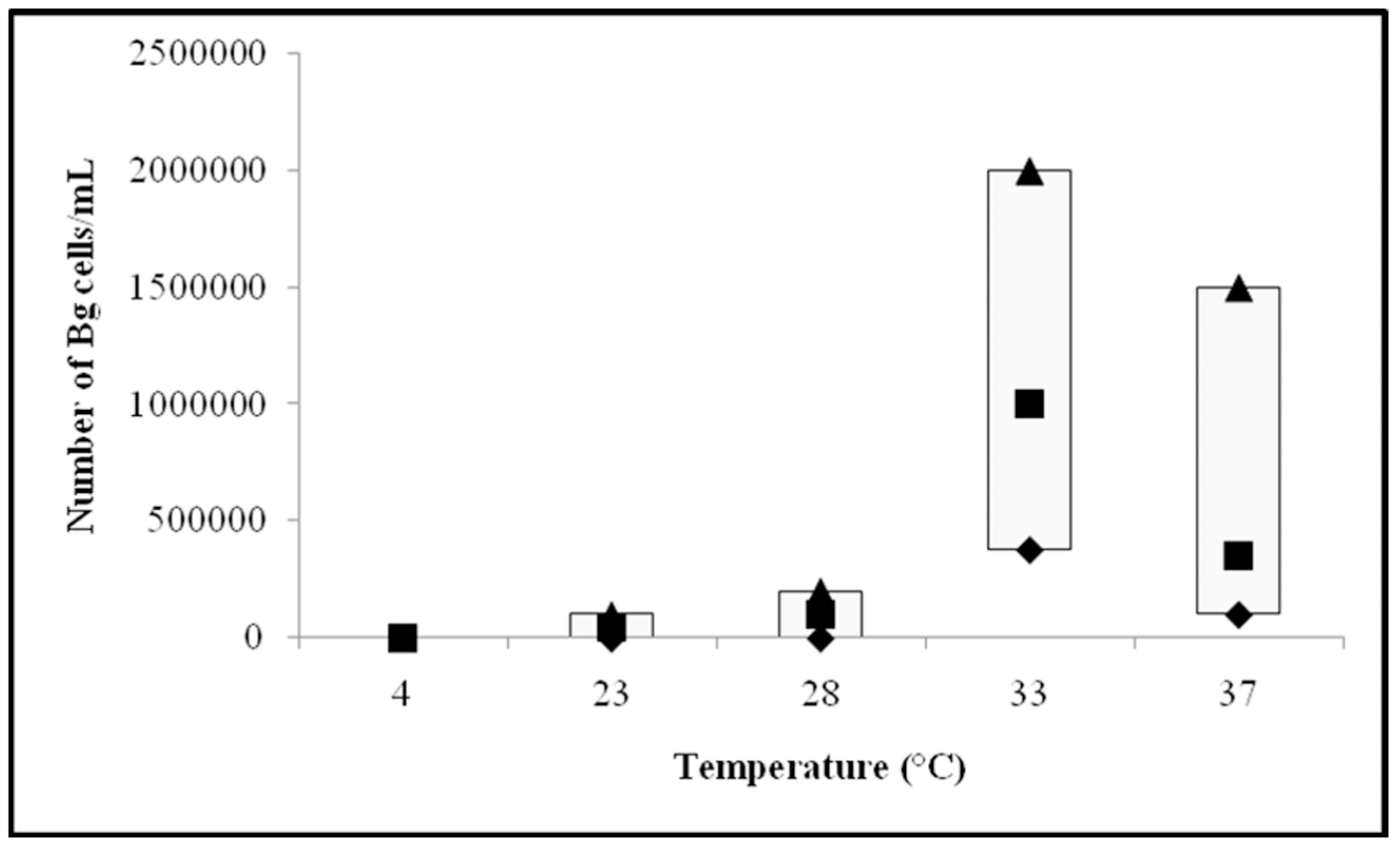
Growth of *Borrelia garinii* (Bg) strains at 4, 23, 28, 33 and 37°C. Median (■), P25 (♦) and P75 (▲) are expressed as number of cells/mL.

**Fig 3:**
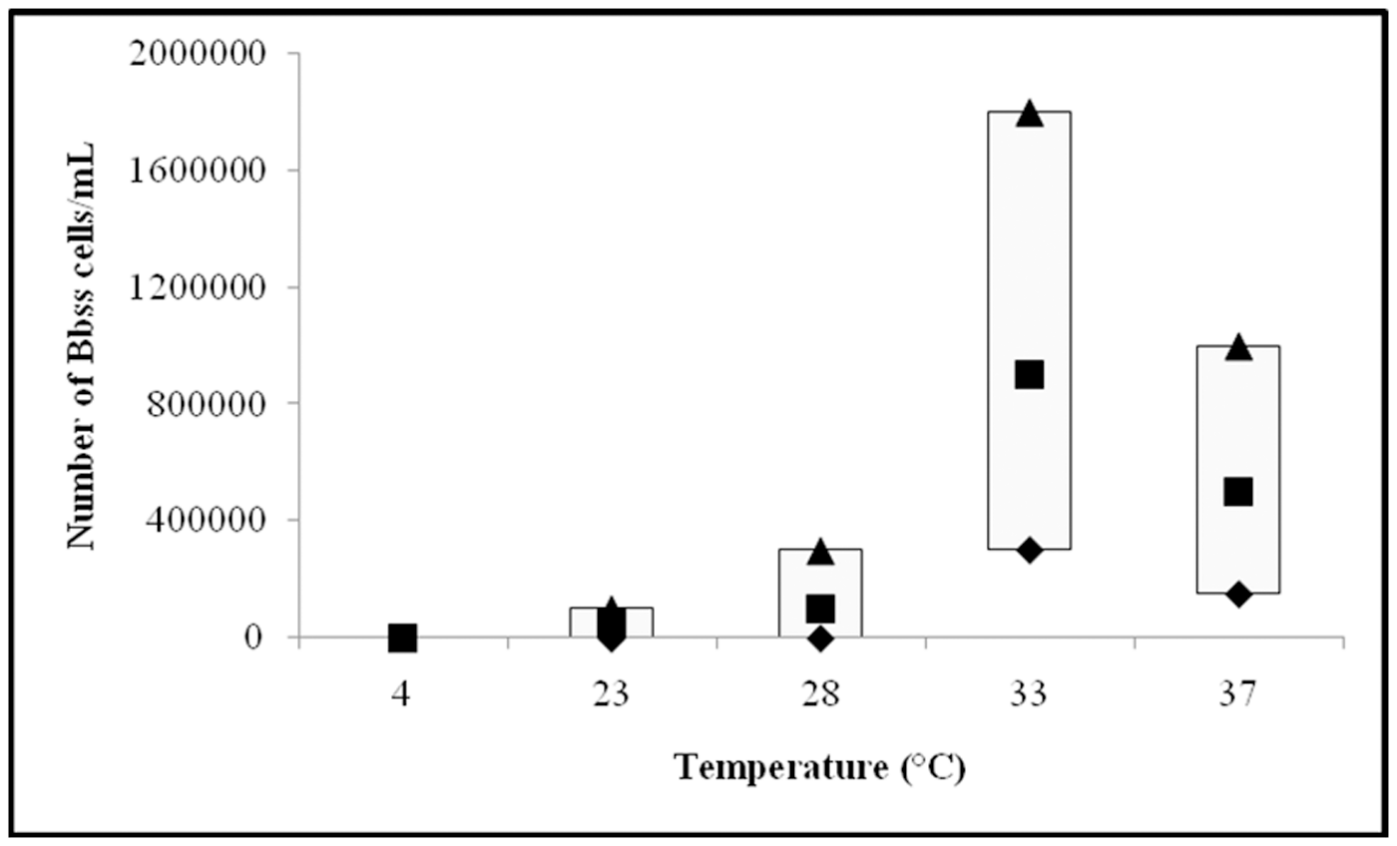
Growth of *Borrelia burgdorferi* sensu stricto (Bbss) strains at 4, 23, 28, 33 and 37°C. Median (■), P25 (♦) and P75 (▲) are expressed as number of cells/mL.

Growth of individual *Borrelia* species at particular temperature is shown on Figs 1–3.

#### *B*. *afzelii* strains

Comparison of the growth of 10 *B*. *afzelii* strains is presented on Fig 1. The median, P25 and P75 final number of cells/mL for these strains after three days of incubation were as follows: at 4°C median = 0, P25 = 0, and P75 = 0; at 23°C median = 2.5x10^4^, P25 = 0, and P75 = 5x10^4^; at 28°C median = 5x10^4^, P25 = 0, and P75 = 1x10^5^; at 33°C median = 3x10^5^, P25 = 1x10^5^, and P75 = 5x10^5^; and at 37°C median = 1x10^5^, P25 = 0 and P75 = 3x10^5^. Based on final number of *B*. *afzelii* cells, the fastest growth was found at 33°C, followed by 37, 28 and 23°C, while no growth was detected at 4°C (Fig 1); using Kruskal-Wallis test, that compares median values, these differences were statistically significant (*P*<0.001).

The Mann Whitney test that compares growth at two particular temperatures showed statistically significant differences in *B*. *afzelii* growth between particular and all other tested temperatures (*P <*0.05).

#### *B*. *garinii* strains

Comparison of the growth of 10 *B*.*garinii* strains is shown on Fig 2. The median, P25 and P75 final number of 10 *B*. *garinii* strains expressed in cells/mL were as follows: incubation at 4°C median = 0, P25 = 0 and P75 = 0; at 23°C median = 5x10^4^, P25 = 0, and P75 = 1x10^5^; at 28°C median = 1x10^5^, P25 = 0, and P75 = 2x10^5^; at 33°C median = 1x10^6^, P25 = 3.75x10^5^, and P75 = 2x10^6^; and at 37°C median = 3.5x10^5^, P25 = 1x10^5^, and P75 = 1.5x10^6^. Based on final number of *B*. *garinii* cells, the most successful growth was found at 33°C, followed by 37, 28 and 23°C, while no growth was detected at 4°C (Fig 2); using Kruskal-Wallis test for comparison of the median values, these differences were statistically significant (*P*<0.001).

Comparison of *B*. *garinii* growth at two particular temperatures with Mann Whitney test showed statistically significant differences in growth between particular and all other tested temperatures (*P <*0.05).

#### *B*. *burgdorferi* sensu stricto strains

Comparison of the growth of 11 *B*. *burgdorferi* sensu stricto strains is depicted on Fig 3. The median, P25 and P75 final number presented in cells/mL were as follows: at 4°C median = 0, P25 = 0, and P75 = 0; at 23°C median = 5x10^4^, P25 = 0, and P75 = 1x10^5^; at 28°C median = 1x10^5^, P25 = 0, and P75 = 3x10^5^; at 33°C median = 9x10^5^, P25 = 3x10^5^, and P75 = 1.8x10^6^; and at 37°C median = 5x10^5^, P25 = 1.5x10^5^, and P75 = 1x10^6^. Based on final number of borrelia cells, the best growth was found at 33°C, followed by 37, 28 and 23°C, while no growth was detected at 4°C (Fig 3); comparison of the median values (Kruskal-Wallis test) showed that the differences were statistically significant (*P*<0.001). Statistically significant differences were found also comparing the gowth of *B*. *burgdorferi* sensu stricto strains at different temperatures (*P <*0.05).

### Comparison of growth at particular temperatures according to *Borrelia* species (*B*. *afzelii*, *B*. *garinii*, and *B*. *burgdorferi* sensu stricto)

**Fig 4:**
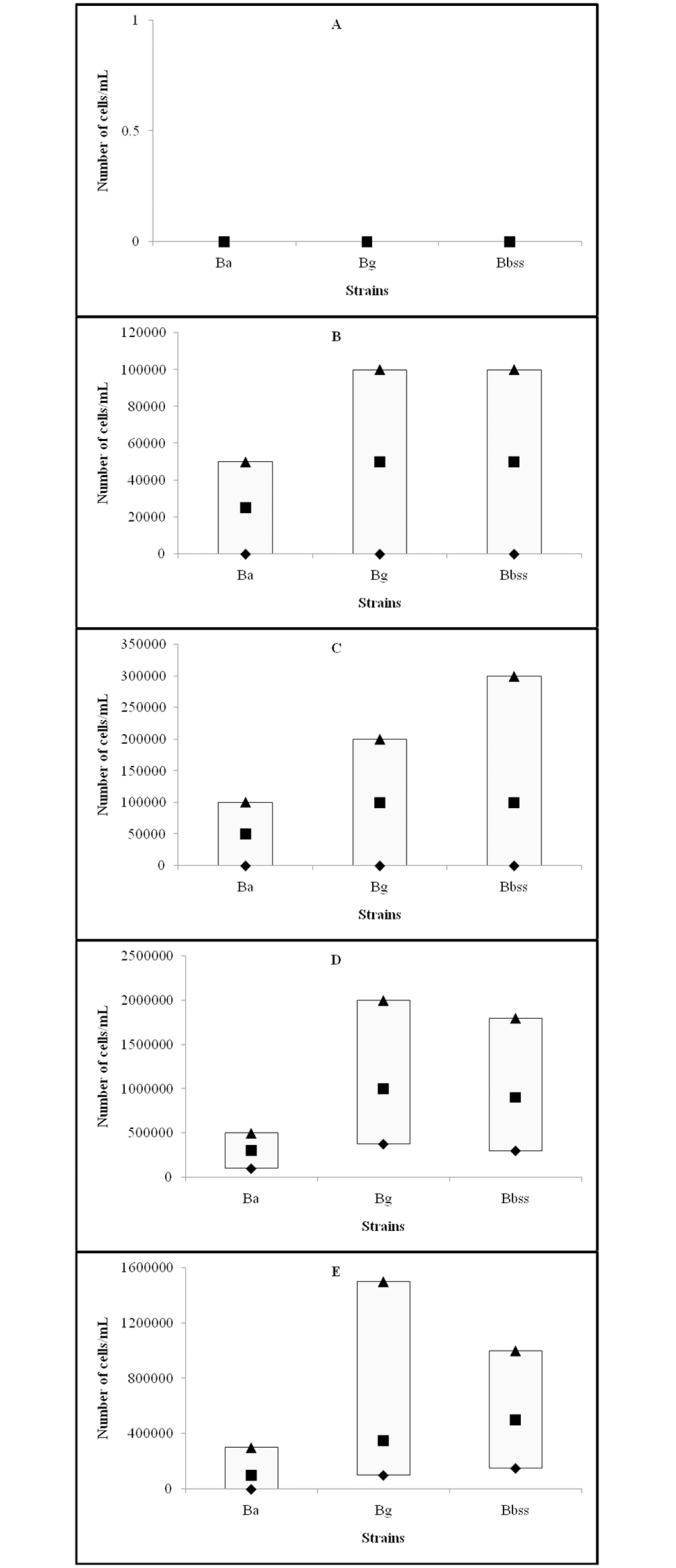
Growth of *Borrelia afzelii*, *Borrelia garinii*, and *Borrelia burgdorferi* sensu stricto at different temperatures expressed as number of *Borrelia* cells/mL and presented as median (■), P25 (♦) and P75 (▲); A: growth at 4°C, B: 23°C, C: 28°C D: 33°C and E: 37°C.

Comparison of growth of *B*. *afzelii*, *B*. *garinii*, and *B*. *burgdorferi* sensu stricto strains at different temperatures is shown on Fig 4A–4E.

#### Comparison of growth at 4°C

The median, P25, and P75 final number (in cells/mL) of all three *Borrelia* species (*B*.*afzelii*, *B*. *garinii*, *and B*. *burgdorferi* sensu stricto) were 0; the Kruskal-Wallis test did not show statistically significant differences in growth at 4°C between the three *Borrelia* species (*P* = 0.263). Results are presented on Fig 4A.

#### Comparison of growth at 23°C

The median, P25, and P75 final number (in cells/mL) of three *Borrelia* species at 23°C were as follows: *B*. *afzelii* median = 2.5x10^4^, P25 = 0 and P75 = 5x10^4^; *B*. *garinii* median = 5x10^4^, P25 = 0, and P75 = 1x10^5^; and *B*. *burgdorferi* sensu stricto median = 5x10^4^, P25 = 0, and P75 = 1x10^5^, respectively. Results are presented on Fig 4B.

The Kruskal-Wallis test showed statistically significant differences in the growth at 23°C between *B*. *afzelii*, *B*. *garinii*, and *B*. *burgdorferi* sensu stricto after three days of incubation (*P* = 0.021).

The Mann-Whitney test demonstrated statistically significant differences between the growth of *B*. *afzelii* and *B*. *garinii* strains (*P* = 0.023), as well as between *B*. *afzelii* and *B*. *burgdorferi* sensu stricto strains (*P* = 0.001), while no statistically significant difference was found comparing the growth of *B*. *garinii* and *B*. *burgdorferi* sensu stricto strains (*P* = 0.807).

#### Comparison of growth at 28°C

The median, P25, and P75 final number (in cells/mL) of three *Borrelia* species at 28°C were as follows: *B*. *afzelii* median = 5x10^4^, P25 = 0, and P75 = 1x10^5^; *B*. *garinii* median = 1x10^5^, P25 = 0 and P75 = 2x10^5^; and *B*.*burgdorferi* sensu stricto median = 1x10^5^, P25 = 0 and P75 = 3x10^5^ (Fig 4C).

The Kruskal-Wallis test showed statistically significant differences in the growth at 28°C between three *Borrelia* species (*B*. *afzelii*, *B*. *garinii*, and *B*. *burgdorferi* sensu stricto) after three days of incubation (*P*<0.001).

The Mann-Whitney test demonstrated statistically significant difference between the growth of *B*. *afzelii* and *B*. *garinii* (*P* = 0.014) as well as comparing the growth of *B*. *afzelii* and *B*. *burgdorferi* sensu stricto (*P*<0.001) strains, while comparison of *B*. *garinii* and *B*. *burgdorferi* sensu stricto strains revealed no significant difference in the growth (*P* = 0.123).

#### Comparison of growth at 33°C

The median, P25, and P75 final number (in cells/mL) of three *Borrelia* species at 33°C were as follows: *B*. *afzelii* median = 3x10^5^, P25 = 1x10^5^, and P75 = 5x10^5^; *B*. *garinii* median = 1x10^6^, P25 = 3.75x10^5^, and P75 = 2x10^6^; and *B*. *burgdorferi* sensu stricto median = 9x10^5^, P25 = 3x10^5^ and P75 = 1.8x10^6^, respectively. Results are presented on Fig 4D.

The Kruskal-Wallis test showed statistically significant differences in growth at 33°C between three *Borrelia* species (*B*. *afzelii*, *B*. *garinii*, and *B*. *burgdorferi* sensu stricto) after three days of incubation (*P*<0.001).

The Mann-Whitney test demonstrated statistically significant difference between the growth of *B*. *afzelii* and *B*. *garinii* (*P*<0.001) as well as *B*. *burgdorferi* sensu stricto (*P*<0.001), while no significant difference was found between *B*. *garinii* and *B*. *burgdorferi* sensu stricto (*P* = 0.868).

#### Comparison of growth at 37°C

The median, P25 and P75 final number (in cells/mL) of three *Borrelia* species at 33°C were as follows: *B*. *afzelii* median = 1x10^5^, P25 = 0, and P75 = 3x10^5^; *B*. *garinii* median = 3.5x10^5^, P25 = 1x10^5^, and P75 = 1.5x10^6^; and *B*. *burgdorferi* sensu stricto median = 5x10^5^, P25 = 1.5x10^5^, and P75 = 1x10^6^, respectively. Results are presented on Fig 4E.

The Kruskal-Wallis test showed statistically significant differences in growthat 37°C between three *Borrelia* species (*B*. *afzelii*, *B*. *garinii*, and *B*. *burgdorferi* sensu stricto) after three days of incubation (*P*<0.001).

The Mann-Whitney test demonstrated statistically significant difference comparing the growth of *B*. *afzelii* and *B*. *garinii* (*P*<0.001) as well as *B*. *afzelii* and *B*. *burgdorferi* sensu stricto strains (*P*<0.001), while no significant difference was found comparing the growth of *B*. *garinii* and *B*. *burgdorferi* sensu stricto strains (*P* = 0.490).

## Discussion

Several factors influence *in vitro* borrelia growth such as medium ingredients and its pH, temperature of incubation, the presence of contaminants, sample’s cell density, number of borrelia strains in the sample, antecedent antibiotic therapy, capacity of particular *Borrelia* species to grow, etc. [10, 12, 14, 22, 24, 25]. Since there are many differences between bacterial growth *in vivo* and *in vitro*, the *in vitro* conditions and consequently the findings of the *in vivo* studies are only a rough approximation of the *in vivo* situation [26]. Therefore, the results of the *in vitro* studies can be used as a basis for understanding of the *in vivo* events but not as a proof that *in vitro* and *in vivo* findings are equivalent.

In the present study, we analyzed *in vitro* growth of 10 *B*. *afzelii*, 10 *B*. *garinii*, and 11 *B*. *burgdorferi* sensu stricto strains at different temperatures within the temperature range from 4 to 37°C. Even though similar *in vitro* studies have been reported previously [12, 13] their findings were limited due to a very small number of examined strains.

Temperature is one of the most important factors influencing bacterial growth. The optimal temperature for borrelia *in vitro* growth was reported to be in a rather wide range from 30 to 37°C [10]; borreliae are normally cultivated at temperatures 30 to 34°C, most often at 32 to 33°C [9, 13, 14, 16, 18, 22, 27].

Hubálek et al. [12] reported on differences in optimal growth temperatures for distinct *Borrelia* species. The authors tested only three borrelia strains (one *B*. *afzelii*, *B*. *garinii* and *B*. *burgdorferi* sensu stricto, respectively) and found that the optimal temperature for strain B31 (*B*. *burgdorferi* sensu stricto) was 33°C, for strain BR75 *(B*. *afzelii*) 35°C, and for strain BR14 (*B*. *garinii*) 37°C. Heroldová et al. [13] tested the same B31 strain and confirmed that 33°C is the optimal growth temperature for the strain, but the strain showed good growth also at 28, 30, 35 and 37°C. Since results based on one strain may not represent a typical finding for individual *Borrelia* species we analyzed larger number of strains that included 10 *B*. *afzelii*, 10 *B*. *garinii*, and 11 *B*. *burgdorferi* sensu stricto strains (Table 1). The best growth for all three *Borrelia* species was ascertained at 33°C; a good growth was observed at 37°C, while growth was weak at 28 and 23°C, and not detectedat 4°C (Figs 1–3). The finding that all three *Borrelia* species survived at 23, 28, 33, and 37°C during three days' incubation suggests that temperature range 23 to 37°C can be suitable for transport of clinical material to the laboratory and that these temperatures should not have a substantial negative impact on the survival of borreliae in the sample as well as on their further isolation and cultivation.

Among three *Borrelia* species, the growth of *B*. *afzelii* was weaker than the growth of *B*. *garinii* or *B*. *burgdorferi* sensu stricto, while the growth of *B*. *garinii* and *B*. *burgdorferi* sensu stricto was comparable. These findings were valid for temperatures 23, 28, 33 and 37°C. As expected, no growth was observed at 4°C (Fig 4A–4E).

It is well known that *B*. *afzelii* and *B*. *garinii* are mostly associated with skin manifestations (erythema migrans and acrodermatitis chronica atrophicans) and nervous system involvement (Lyme neuroborreliosis), respectively, while *B*. *burgdorferi* sensu stricto is associated with joint involvement (Lyme arthritis), which is more often observed in North America than in Europe [1, 2]. Some authors suggested that differences in *Borrelia* species organotropism may be due to distinct tissue temperature [11, 12]. Hubalek et al. [12] tested *in vitro* growth of three different pathogenic *Borrelia* species at different temperatures and showed that *B*. *garinii* was the most thermotolerant species, followed by *B*. *afzelii*, while *B*. *burgdorferi* sensu stricto was the least theromtolerant. Thus, “heat-stable” *Borrelia* species affect warmer regions of the body (e.g. *B*. *garinii* is mostly associated with Lyme neuroborreliosis), while “heat-sensitive” borrelia strains involve body regions with lower temperature (e.g. *B*. *afzelii* is mostly associated with skin manifestations) [11]. However, the results of the present study do not support previous hypothesis that different *Borrelia* species organotropism could be a consequence of distinct tissue temperature.

Generally, difference in growth between borrelia strains at different temperatures may be interpreted as natural borrelia characteristic.

We demonstrated that the initial number of borrelia cells/mL had statistically significant positive influence on the growth of all three *Borrelia* species (*B*. *afzelii*, *B*. *garinii*, and *B*. *burgdorferi* sensu stricto) at all tested temperatures, except at 4°C (Table 3). This findings suggest that a large number of borrelia cells/mL present in a clinical sample has good chances to survive, adapt and grow during *in vitro* incubation while specimen with small number of borrelia/mL may adversely influence borrelial isolation rate and the outcome of cultivation [14, 22].

## Conclusions

Our study revealed that 23, 28, 33 and 37°C were suitable for borrelia growth and survival. For all three *Borrelia* species (*B*. *afzelii*, *B*. *garinii*, and *B*. *burgdorferi* sensu stricto), the optimal temperature was 33°C, followed by 37, 28, and 23°C. There was no statistical significant difference between the growth of *B*. *garinii* and *B*. *burgdorferi* sensu stricto at 23, 28, 33 and 37°C, respectively, while the growth of *B*. *afzelii* species was weaker in comparison to the other two species. The initial number of borrelia cells/mL influenced on growth of all three *Borrelia* species at all tested temperatures, except at 4°C, suggesting that the large number of borrelia is important for successful cultivation. Reported difference in growth of borrelia strains at different temperature may be interpreted as natural borrelia characteristic.
